# Barriers and facilitators of the HIV care continuum in Southern New England for people with drug or alcohol use and living with HIV/AIDS: perspectives of HIV surveillance experts and service providers

**DOI:** 10.1186/s13722-017-0088-7

**Published:** 2017-10-02

**Authors:** Lauretta E. Grau, Abbie Griffiths-Kundishora, Robert Heimer, Marguerite Hutcheson, Amy Nunn, Caitlin Towey, Thomas J. Stopka

**Affiliations:** 1 Yale School of Public Health, PO Box 208034, New Haven, CT 06520-8034 USA; 2 Tufts University School of Medicine, Boston, MA 02111 USA; 3 Brown University School of Public Health, Providence, RI 02912 USA

**Keywords:** HIV/AIDS, Substance use, HIV care continuum

## Abstract

**Background:**

Contemporary studies about HIV care continuum (HCC) outcomes within substance using populations primarily focus on individual risk factors rather than provider- or systems-level influences. Over 25% of people living with HIV (PLWH) have substance use disorders that can alter their path through the HCC. As part of a study of HCC outcomes in nine small cities in Southern New England (population 100,000–200,000 and relatively high HIV prevalence particularly among substance users), this qualitative analysis sought to understand public health staff and HIV service providers’ perspectives on how substance use may influence HCC outcomes.

**Methods:**

Interviews with 49 participants, collected between November 2015 and June 2016, were analyzed thematically using a modified social ecological model as the conceptual framework and codes for substance use, HCC barriers and facilitators, successes and failures of initiatives targeting the HCC, and criminal justice issues.

**Results:**

Eight themes were identified concerning the impact of substance use on HCC outcomes. At the individual level, these included coping and satisfying basic needs and could influence all HCC steps (i.e., testing, treatment linkage, adherence, and retention, and viral load suppression). The interpersonal level themes included stigma issues and providers’ cultural competence and treatment attitudes and primarily influenced treatment linkage, retention, and viral load suppression. These same HCC steps were influenced at the health care systems level by organizations’ physical environment and resources as well as intra-/inter-agency communication. Testing and retention were the most likely steps to affect at the policy/society level, and the themes included opposition within an organization or community, and activities with unintended consequences.

**Conclusions:**

The most substantial HCC challenges for PLWH with substance use problems included linking and retaining in treatment those with multiple co-morbidities and meeting their basic living needs. Recommendations to improve HCC outcomes for PLWH with substance use problems include increasing easy access to effective drug and mental health treatment, expanding case management and peer navigation services, training staff about harm reduction, de-stigmatizing, and culturally competent approaches to interacting with patients, and increasing information-sharing and service coordination among service providers and the social service and criminal justice systems.

## Background

The HIV care continuum (HCC) framework assesses patients at various steps of human immunodeficiency virus (HIV) diagnosis and care—from identification of cases, to linkage to care and antiretroviral treatment, to retention in care, and ultimately to viral suppression [1]. Each step builds upon the previous, and the proportion of PLWH within each step has important implications for achieving the ultimate goals of viral suppression and reduced HIV transmission [2]. Monitoring the outcome at each HCC step enables us to better identify where and how to intervene—be it a specific HCC step, geographic area, or at-risk population. The HCC is also a tool by which to monitor the UNAIDS 90-90-90 goal of identifying 90% of those infected, linking 90% of those identified to treatment, and achieving 90% viral suppression among those in treatment; it is believed that reaching this goal by 2020 would end the HIV epidemic by 2030 [3].

Despite reported overall improvements in HCC outcomes [4–6], negative associations between substance use and virtually every step on the continuum persist [7–10]. And although medical management of a patient’s HIV infection and substance use problems can be complex [11], medication-assisted treatment improved HCC outcomes for PLWH with opioid use disorders [12] and decreased injection risk behavior [13]. Nonetheless, PLWH with substance use problems do not fare as well as other risk groups [4, 9, 14]. PLWH who used substances intermittently or continually were significantly more likely to develop opportunistic infections or experience disease progression or mortality when compared to PLWH with no reported substance use [15]. Substance use problems interfered with progression along the HCC for female PLWH; its treatment and that of related co-morbidities (e.g., depression) could help increase retention in care [16]. In addition to the potential instability that substance use can bring to the lives of PLWH, other sources of instability (i.e., financial, homelessness, housing insecurity, stigma, and food insecurity) also influence HCC retention rates for these individuals [17–19].

It is estimated that over one-quarter of PLWH have substance use disorders [20]. Recent studies have primarily focused on individual risk factors [21–24]. To our knowledge, there is scant information in the literature that examines HIV provider perspectives on the role of substance use in HCC outcomes, how structural or provider factors may influence progression on the continuum for those who use substances, or the role of substance use in continuum outcomes in smaller urban areas. Structural influences such as organizational, social, policy, or economic factors can include the convenience of access to and the array of HIV-related services offered, confidentiality issues, or the existence of laws that discriminate against marginalized populations such as people with substance use problems, commercial sex workers, or undocumented immigrants or seasonal workers. Provider influences can include provider attributes such as provider-initiated HIV testing and counseling as a strong motivator to be tested [25, 26], prescribers’ opinions about when to initiate antiretroviral treatment [14], interpersonal skills, and cultural competence and can alter the path of PLWH at various steps of the HCC.

Several initiatives have helped to reduce the negative impact of substance use on HIV prevention, diagnosis, and treatment [27]. These include drug treatment programs that routinely test for HIV [28] and provide HIV prevention education [28]. Harm reduction programs such as syringe services programs (SSPs) that distribute condoms [29, 30] and provide access to testing and HIV and substance abuse treatment have also been demonstrated to be effective [31–38]. Evidence-based interventions that promote linkage and retention, such as case management, improved screening for substance used disorders and mental health, and peer navigators can be effective in improving retention rates [19].

Most HCC research has occurred in large urban and metropolitan areas [39, 40], with less known about HCC outcomes in smaller urban areas where a large share of new HIV infections occur. Structural and sociodemographic factors and geographic access to local resources differ between these larger urban areas and smaller cities and can also vary across smaller cities [41]. In Southern New England, thousands remain undiagnosed, and known cases have been lost to follow-up, thereby jeopardizing their own health and the health of the larger community. Within Connecticut, 8239 of 10,636 PLWH (77%) were engaged in care through 2014. Of those engaged, 81% were retained in care, and 86% were virally suppressed [42]. Among the nearly 20,000 PLWH in Massachusetts as of 2015, 75% were engaged in care, 59% were retained in care, and 28% did not have a viral load test through 2014. Among those who had a viral load test, 65% were virally suppressed, with even lower rates of viral suppression in Western Massachusetts [43]. The number of new HIV diagnoses in Rhode Island increased 33% from 2013 to 2014; 67% of PLWH were engaged in care, 48% were retained in care, and 57% were virally suppressed [44].

This qualitative study was part of a larger pilot project that explored the geographic differences in HCC outcomes in nine small cities in Southern New England (Lowell, Springfield, Worcester, New Bedford, Providence, Hartford, Waterbury, New Haven, and Bridgeport) and addressed the funders’ interest in conducting HCC research in small cities across Southern New England. The current analysis used key informant (KI) interviews with HIV service providers and public health staff to understand how substance use problems shape HCC outcomes and to identify potential recommendations to promote receipt of HIV diagnosis, linkage to and retention in care, and viral suppression among PLWH with drug or alcohol problems. Given the time and resource limits of the pilot study, however, we sought to assess HCC outcomes from the multiple perspectives of these individuals (cf., interviews with PLWH) based on our assumption that their knowledge about the existing structural and organizational factors that may influence HCC outcomes would inform a less commonly studied research area and have important implications for future interventions to improve HCC outcomes.

## Methods

### City selection

We included cities with populations of 100,000–200,000 and relatively high HIV prevalence, particularly among people who use drugs and men who have sex with men (MSM). In addition, the cities differed in terms of how they have responded to the HIV epidemic, with only some implementing SSPs.

### Study sample

Using a purposive sampling strategy that targeted staff with HCC-associated responsibilities (e.g., service provision, surveillance, monitoring), potential participants were initially identified through recommendations by state public health officials, an internet search of HIV service organizations in the nine cities, and purposive and snowball sampling of the authors’ colleagues working in the HIV field who satisfied the inclusion criteria. Given the extent of the authors’ (LG, RH, AN, TS) previous HIV and harm reduction research, many participants had established relationships with these individuals. Enrolled subjects subsequently referred us to other potential subjects.

Potential subjects were contacted by telephone or email and, if eligible, were scheduled for an interview. Inclusion criteria were: (1) individuals working in or responsible for HCC-related work in one of the nine cities (e.g., HIV surveillance staff at state or local health departments, Ryan White policy makers or administrators, HIV-associated service providers such as health care providers, early intervention specialists, disease intervention specialists, case managers, social workers, peer navigators); (2) at least five years of experience in the HIV field; and (3) English speaking. There were no refusals to participate among those who were eligible for the study.

We interviewed 49 participants between November 2015 and June 2016; there were no follow-up interviews. All were scheduled as individual, face-to-face interviews with trained interviewers (LG, MH, AK, CT, TS). One interviewer was male; all held advanced degrees in the fields of public health or social science or had previous qualitative research experience. Seven KIs had invited other staff members to join based upon the belief that those other people could offer additional information about one of the interview domains. We honored the original KI’s decision and confirmed that these additional KIs satisfied inclusion criteria. The interviews lasted approximately 60 min, were digitally recorded, and subsequently transcribed verbatim (transcripts were not reviewed by participants). The study was deemed exempt from human subjects research by the IRBs at Yale, Tufts, and Brown University. The interviews began after an informed consent discussion, and participants received a $25 gift card as reimbursement for the interview.

### Data collection and analysis

The entire research team developed and reviewed a draft interview guide that included the five HCC steps as interview domains and assessed participants’ perceptions about the specific successes and challenges encountered for each step (i.e., HIV testing, treatment linkage, initiation of antiretroviral therapy, treatment retention, and viral suppression). Probes for each HCC step included asking about the perceived *availability* and *accessibility* of HIV-associated services, treatment *acceptability* by PLWH, and *affordability* of such care [45].

While the five HCC steps served to structure and organize the flow and topics covered during the interviews, we used a modified social ecological model as the conceptual framework by which to organize the salient themes about the relationship between substance use and HCC outcomes [2, 46, 47]. The modified social ecological model is a multilevel model that situates the individual within the social and structural context when examining health outcomes [46]. The individual level includes demographic, biologic, and behavioral factors and intrapsychic factors such as self-efficacy and motivation. The individual level is contained within the social or interpersonal level which, in turn, is contained within the community level (which focused exclusively on the health and social service systems in the current analysis), and all are contained within the society or policy level. The latter two levels focus on structural factors.

Once transcripts were available, the coding team (LG, AK, MH) independently coded six transcripts and met weekly to discuss coding decisions and develop the codebook. Any coding discrepancies were resolved by consensus. Additional codes and further refinement of the codebook also occurred during these sessions. When no new codes were identified (i.e., thematic saturation was achieved) and acceptable inter-coder reliability had been achieved, the remaining transcripts were independently coded (by AK or MH) and reviewed by the first author when entering the data into ATLAS.ti (Version 7.1.7). Codes pertaining to substance use, HCC barriers and facilitators, successes and failures of initiatives targeting the HCC, and criminal justice issues were analyzed thematically [48, 49] in an iterative fashion, using an inductive approach wherein themes were grounded in the data. The coding team identified common patterns across the dataset, grouped them into themes, and sought negative instances where the data did not fit the existing themes as part of the confirmability process [50]. To improve readability without compromising content, all colloquialisms, hesitations, and non-verbal utterances were removed from the quotes. Participants did not review the analytic findings.

**Fig. 1:**
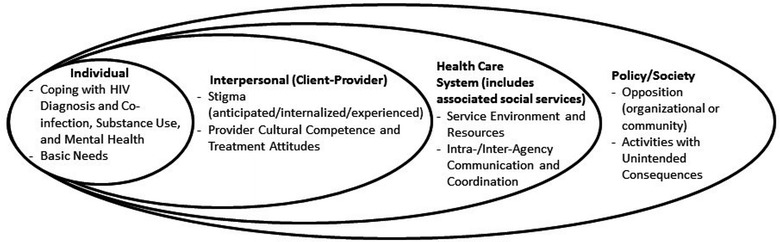
Modified social ecological model for HIV treatment

Themes were grouped by level within the modified social ecological model and specifically focused on the impact of substance use on HCC outcomes. Hence, the interpersonal level themes were restricted to those that shaped the client-provider relationship or motivation to seek testing or treatment and community level themes to those involving care for PLWH. A total of nine themes were identified across the four levels of the modified social ecological (Fig. 1). It should be noted that the influence of each theme on the HCC could be positive or negative, depending upon the degree to which each is present or absent.

## Results

### Description of the study sample

The majority of participants held supervisory or administrative positions within AIDS service organizations (ASOs) and had detailed knowledge about the types and quality of local services available (Table 1). Interviews with regional Ryan White administrators and state-level epidemiologists and program managers provided a broader, “bird’s eye view” of the HCC and local services. Interviews with local medical providers (16%) and caseworkers (12%) provided examples of HCC successes and failures from participants’ experiences working with PLWH with substance use problems.

**Table 1:** Description of study sample and interview types

Interview characteristics	Participants no. (%)
State (N = 44 interviews)	
Connecticut	18 (41)
Massachusetts	21 (48)
Rhode Island	5 (11)
Interviews per city (N = 38)	
Bridgeport, CT	4 (10.5)
Hartford, CT	4 (10.5)
New Haven, CT	4 (10.5)
Waterbury, CT	4 (10.5)
Lowell, MA	5 (13)
New Bedford, MA	4 (10.5)
Springfield, MA	6 (16)
Worcester, MA	4 (10.5)
Providence, RI	3 (8)
Organization type (N = 44 interviews)^a^	
State Health Department	5 (11)
Local Health Department	3 (7)
Ryan White (local and regional)	4 (1)
AIDS Service Organization	36 (82)
Staff type (N = 49 participants)^a^	
Regional/state program administration	6 (12)
Local administrative/supervisory	31 (63)
Medical providers (MD/APRN)	8 (16)
Case management/EIS/DIS	6 (12)
Sex (N = 49 participants)	
Female	23 (47)
Male	26 (53)

A total of 49 individuals participated in the interviews

^a^Some interviews involved participants with multiple job responsibilities

### Successful initiatives to improve the HCC

Before identifying the themes about how substance use problems shape HCC outcomes, it is important to acknowledge the improvements that have already been achieved in reducing the negative impact of drug and alcohol use on HCC outcomes. These included reductions in HIV incidence in conjunction with HIV testing and prevention efforts at SSPs, community expansion of medication-assisted treatment, and efforts within the Department of Corrections. A medical provider acknowledged that expanded syringe access through SSPs and pharmacy sales has *“kind of done it already [fewer HIV cases among entering inmates within the Department of Corrections] with injection drug use.”* Others noted the community’s belief in the public health benefits of SSPs, in one case assuming operation of the SSP when city officials wanted to discontinue it.

We don’t see as many intravenous drug users getting infected as we did, obviously, because they have access to syringes. (Participant 13, Male, ASO administrator)

And [the community has] come together to do the [SSP] van and to expand services going—that’s the heart that exists here. There was no turf. It’s like we need, for our folks, we’re going to do what it takes. (Participant 11, Male, Ryan White administrator)

Efforts within the criminal justice system have also improved HCC outcomes by identifying new infections or resuming treatment among entering inmates who had been lost to treatment. These advances were acknowledged by virtually all participants with knowledge about Department of Corrections activities or data.

We are not testing nearly as much as we used to at the DoC, and that’s mostly because the number of positives dropped dramatically when the epidemic shifted away from injection drug use. (Participant 39, Male, Medical Provider)

### Individual level themes

The advances noted above, notwithstanding, PLWH who use drugs or alcohol continue to face challenges that can affect HCC outcomes. These include the individual’s coping style, comorbid conditions (e.g., hepatitis C virus co-infection, substance abuse, mental health), and resources to meet basic needs (e.g., housing, food security, transportation, clothing, childcare).

#### Coping with HIV diagnosis and comorbidities

Receiving an HIV diagnosis, being co-infected, or having mental health problems (whether associated with or independent of the HIV diagnosis) can be a source of stress that some PLWH may attempt to cope with by self-medicating with alcohol or drugs. Ongoing substance use problems can, in turn, pose significant barriers to the HCC steps of treatment linkage, adherence, and viral suppression. Many identified the need for mental health services to address either the co-morbid condition or maladaptive coping style.

Mental health is a big one. “I’m depressed; therefore, I’m not taking it [HIV medications].” (Participant 1, Female, ASO administrator)

A lot of times what we’ve found is it’s people with mental health issues trying to make themselves feel better by using substance. (Participant 9, Male, ASO administrator)

How problems with substance use and co-morbidities manifest can be highly idiosyncratic. Many participants reported that clients with ongoing substance use or who had experienced relapse—particularly with opioids—often stopped receiving their regular HIV care. By contrast, a few participants observed that some PLWH remained highly engaged in their treatment despite using drugs or alcohol. The possible reasons for these observed differences remained unclear, however.

[HIV infection is] a tremendous psychological stress that people carry with them day in, day out, and some people respond well to it and some don’t. (Participant 39, Male, Medical Provider) Some people end up, when they’re actively using drugs, [they] are out there and not taking meds and not keeping their appointments, and just disappear and have a whole other mode of circling and who they’re seeing. And on the other hand we have folks that are actively using heroin and have a pretty stable pattern of life and [are] coming in for their visits and taking all their meds and, and the heroin is not destabilizing. (Participant 31, Male, Medical Provider)

And I think that even those individuals who are in care who are using…I think they do not want to do harm to their families or the women and/or men in their lives. I think they want to make sure that they are protecting them and their selves, and so I think that has all to do with the retention rate. (Participant 15, Female, City Health Department)

Inherent in attempts to cope with a given health problem is the level of motivation to seek help. Although less frequently mentioned, motivation to enter mental health or substance abuse treatment was viewed as critical to successful HIV treatment linkage, retention, and adherence. Participants noted that for substance abuse problems in particular, bureaucratic hassles and ineffective treatments can seriously reduce the chances of entry into treatment that could mitigate substance use problems that interfere with HIV treatment adherence.

With substance abuse, there has to be a desire to actually want to get help. I would let them know where those resources are, but I can’t like physically force them to go. (Participant 5, Female, Case worker)

You want to get in the detox because you’re finally sick of drinking and using drugs, and it’s such a dehumanizing process. You go through this long intake and then they say, “Oh, we don’t take your insurance, try this other place,” or “Okay, we’re going to put you on the waiting list. You’ll have to call us three times a day to find out if we get a bed open.” And it’s days and usually by the time a bed does open, so many people have lost their motivation and just said, “Screw it, I’m going to go out and get another bag of dope.” (Participant 12, Female, ASO administrator)

[Detox is] a band-aid. It doesn’t—it has yet to work once since I’ve been at [this clinic]. (Participant 48, Female, Medical Provider)

#### Fulfilling basic living needs

Basic needs such as housing, food, and transportation, if unmet, can also pose significant challenges to PLWH. Participants reported that these unmet needs can be stressful, leading to substance use or relapse and are often higher priorities than is treatment linkage or adherence. Housing opportunities were a *“major, major issue”* according to participants, and the possibility of stable housing was especially limited for those with substance use problems.

Everything just piggybacks each other, housing, transportation, food, income, medication, doctors’ appointments, everything just piggybacks each other. (Participant 47, Female, Case worker)

But it’s very hard to place somebody in housing that doesn’t have a job or somebody who is multiple evictions. Those things are still factors in placing folks in housing. Somebody with significant or severe mental health and substance abuse challenges, those things still are challenges for people accessing stable housing. (Participant 18, Female, Ryan White administrator)

I mean, the care of HIV itself is not the problem. It’s just all of the other social situations surrounding it that make it challenging. So we have one patient that I think I saw last who came in here today who is living in her car. (Participant 24, Female, ASO administrator)

Somebody might let the medicines go while they’re working on the housing. (Participant 31, Male, Medical Provider)

### Interpersonal level themes

#### Stigma

PLWH are susceptible to three types of stigma: (1) enacted stigma or individuals’ belief that they had a stigmatizing experience associated with their having HIV infection, mental health problems, or drug or alcohol use, (2) anticipated stigma or individuals’ expectation of experiencing stigma in some future situation or context, and (3) internalized stigma or the degree to which individuals endorse the negative attributes associated with one or all of these health problems [51]. Although many acknowledged that HIV stigma has decreased over time, participants primarily discussed stigma in terms of anticipated HIV stigma within the clients’ communities (most notably immigrant or minority communities) and far less frequently from service providers. The anticipated stigma which could impact HCC outcomes was most frequently based on concerns about being stigmatized because of potential confidentiality breaches within these small cities.

There’s stigma about HIV. There’s stigma about mental illness. There’s stigma about addiction. There’s stigma about a number of things. So it seems sort of multi-layered issues. (Participant 39, Male, Medical provider)

Stigma. They don’t want people to see—in a small city, people know each other…That is big. People will see them going into a clinic. People will see them going into an HIV anything, and they don’t want to be seen doing that. (Participant 29, Female, ASO administrator and clinician)

So I think one of the challenges to testing is stigma, and what happens, and who has access to your information, and what… what if my partner finds out? What if my family finds out? (Participant 30, Female, ASO administrator)

I tell my clients right off the bat, because I live in [this city] and I’m in the area and I see a lot of you, when I’m with my family to keep your information confidential and so that your information isn’t breached. When I’m with my family I don’t speak to my clients. (Participant 45, Female, Case worker)

Participants occasionally noted how PLWH may have experienced stigma from their HIV service providers and organizations. It could involve actual experiences with specific providers or how the physical layout of services within the organization could shape stigma perceptions. Of note is that no one mentioned staff training on stigma issues.

I have patients here who say they feel stigmatized every time they’re interacting with their medical provider, their ID specialists. They’re being treated like an addict. (Participant 6, Male, ASO administrator)

Our offices are separate from the adult medicine offices, so even some members of the community, when they come up here, they know if you look to the left, this is where people living with HIV go, and [to the right] is where people who are not living with HIV go. One of our goals is to change that, because, I mean I personally think that it drives stigma. Just the idea that you’re living with HIV, you can’t receive your medical care where everybody else is receiving their medical care. (Participant 42, Male, ASO administrator)

More frequently, participants identified organizational strategies to reduce the potential for stigma. These included giving clinics names that did not identify them as uniquely HIV clinics or incorporating HIV services into non-HIV clinics.

Instead of calling it the HIV department or the infectious disease department, Cardenio (sic) means caring and loving in Spanish…and the thought behind it was the caring, and loving, and compassion that somebody can receive. When we moved to this building, we really had a hard time identifying how do we designate our area, how do people know that they can come to this floor without feeling stigmatized and so it’s our attempt for somebody to be able to come in and say, “I’m here to see the Cardenio (sic) department” versus “I’m here for HIV services.” (Participant 37, Female, ASO administrator)

And in that same vein, to reduce the stigma, we don’t want to be just an HIV testing van, hep C testing van. Our signs are we do blood pressure, glucose screening, so you can all come in. You can get a vaccination. (Participant 28, Female, Medical Provider)

#### Staff cultural competence and treatment attitudes

Positive client-provider relationships were thought to be critical to the success of HIV treatment linkage, adherence, and retention. Although the merits of a harm reduction approach (i.e., “meeting people where they are at”) and staff assuming a respectful, non-judgmental, and supportive stance with their clients are key to positive relationships for any health problem and client population, participants noted that these can be particularly important when interacting with PLWH with drug or alcohol problems. They recognized that shame and guilt issues—about substance use in addition to HIV—can be incapacitating at times. Sensitive and culturally competent interactions, both in the workplace and when staff encounter clients out in the community, are critical to ensuring that clients remain engaged or re-engage in care. However, it should also be noted that discussion about cultural competence most often focused on the number of languages spoken by staff and far less frequently on the attitudes or behaviors that demonstrate cultural competence.

We’re culturally competent here and when we hire staff, we try to get a good array of people that come from different cultures and speak the languages [spoken in this region]. (Participant 2, Female, ASO administrator)

For me, the surface of culturally competent care is care delivered in the language that the person feels most comfortable speaking and understanding. And maybe even the person speaking that language coming from a similar background to the person that’s receiving the education or [care]…So it’s our effort to make sure that we can, at the very least, provide language services…My philosophy, and when I talk to our staff is [that] it’s more about just withholding judgment. I mean, part of being culturally competent is knowing that you can’t be completely culturally competent because if you were, then you’re just basing that competency on norms which don’t apply to everybody. So when people are coming in and talking about certain attitudes and certain beliefs and understandings about the care that they’re coming into access, for me, it’s more about just greeting that understanding with an open mind and trying to understand where it’s coming from and trying to do what you can to address those issues without being, I guess, condescending or without the appearance of being rude or without making the person feel like they are stupid. Which can be complicated because if somebody holds a belief really firmly, and you’re trying to have a discussion with them that sort of is contradicting that belief, then that can be a difficult conversation. (Participant 42, Male, ASO administrator)

Attitudes concerning initiation of antiretroviral therapy varied, particularly for PLWH with drug or alcohol problems, with most stating that it should be initiated as soon as possible. By contrast, providers in one city stated their reluctance to initiate treatment until the patients’ housing, substance use, mental health, or other medical or social service problems were stabilized—some estimating this process could take as long as 6 months.

It just doesn’t make sense to prescribe somebody antiretrovirals and they don’t have a place to live, ya know, or they…they’re actively using drugs and they have uncontrolled schizophrenia, really? It doesn’t make sense, so I really feel strongly you got to address those things first. (Participant 3, Male, Medical Provider)

I have people using crack who have non-detectable viral loads, so it makes me think that they [ART and crack use] can happen at the same time. (Participant 48, Female, Medical Provider)

I’ve known this for years, you got to give them access and availability to a pill, no matter what. (Participant 32, Male, State Health Department)

### Health care systems level themes

Themes at the health care systems level occurred internally within a given organization as well as across organizations and agencies. They were thought to influence HIV treatment, linkage, and retention for people who use drugs or alcohol and included two themes: (1) the organization’s environment such as the services that were available, its physical location and layout, and hours of operation and (2) intra- and inter-agency communications and coordination of services.

#### Service organization environment and resources

Virtually all participants noted the complexity of service needs for PLWH with drug or alcohol problems and agreed that increased availability of and easy accessibility to these needed services was key to improving HCC outcomes. Beyond providing for the basics such as housing, clothing, and food, participants reported that clients often required transportation or daycare services so they could attend their various HIV-related appointments. Clients often needed case management services or other specialty services (e.g., treatment for substance use or mental health problems, hepatitis C virus treatment, physical therapy for motor problems, support and group therapy meetings) or help in managing their finances. The extent to which “one-stop shopping” was possible increased the potential for successful HCC outcomes. Co-locating multiple medical and social services, having flexible office hours, the opportunity for walk-in visits, and being able to address multiple health and social service problems in a single visit facilitated HIV treatment retention and adherence. ASOs that offered on-site services with substance abuse counselors, psychiatric nurses, and/or infectious disease specialists (to treat both HIV and hepatitis C infections) were believed to greatly enhance client care and consequently HCC outcomes. Although medication-assisted treatment services were not co-located at any of the ASOs in this study, referrals to local providers were offered in an attempt to facilitate access to and availability of these services.

[Directly observed therapy] can get done at either place [van or storefront], but usually we like to see them on the van and then we can address any other issues, medical or social, and then we link them either back up to [their case worker] or back up to their primary doctor. And I also can do some primary care with them as well, so they don’t have to try to get an appointment to see their primary medical doctor if I can see them in five minutes—the van is like an urgent care center and it’s kind of, it’s very quick, very fast. (Participant 28, Female, Medical Provider)

And even if I see somebody who might want to have me as a source of their HIV care, I will refer them to the health center, because they’ve got a whole other bucket of needs that I can’t really fulfill, so sort of one-stop shopping. (Participant 43, Male, Medical Provider)

The importance of case management and peer-to-peer programs was repeatedly linked to successful HCC outcomes. It was clear in interviews with medical providers that they respected and viewed case managers, peer navigators, early intervention specialist, and disease intervention specialists as highly knowledgeable about their patients’ current life situations. Providers relied upon these staff for re-engaging clients who use drugs or alcohol—often among the most difficult to retain in care—after missed appointments. Hence, co-location of medical and case management services afforded opportunities for frequent staff interactions and quicker response when patients missed appointments.

It all depends when the doctor calls you and said, “I haven’t seen this patient in a while,” and then I go look for them and then go try to find them. Usually I can find them maybe in a couple hours. Maybe it’ll take me a day—because I already have [an] idea. See, there’s a lot of clients that we see on the van that we already know their routines, so it’s like I walk down the street, like I’ll go from here to downtown, might see ten people living with HIV that I know, so the first thing I ask them, “When was the last time you seen your doctor?” So they said, “Well, I haven’t seen the doctor in six months, I’m having a drinking problem.” I grab the phone, make an appointment…we link them right there. (Participant 33, Male, Case worker)

I was able to hire a nurse and a peer specifically to carry about twenty cases of the most difficult patients to retain in care. And that money is running out [at] the end of July on [that] project, but the state just decided to pick it up because here it’s been extremely—extremely successful. (Participant 13, Male, ASO administrator)

#### Intra- and inter-agency communications and coordination

Most cities attempted to facilitate information-sharing and open communications among HIV service providers in order to best serve their clients. Some accomplished this by having new clients sign consents permitting the organizations to share information between organizations such as medical laboratories, hospitals, social service and medical providers, drug treatment programs, and the Department of Corrections. City-wide meetings also took place to address HCC issues. The local PLWH community participated in some of these meetings in order to empower and engage them in local efforts to improve HCC outcomes and inform service providers about the types of successes, challenges, and recommendations that could guide client-centered and community-engaged prevention and care. Regional Ryan White meetings were another way that providers evaluated and discussed strategies to improve the HCC at the local or regional level. Yet although formal meetings were helpful, it appeared that informal communications were more frequently used to solve problems for individual clients.

The patients already signed a consent to let us work with any of their providers and [access their electronic medical record]. (Participant 28, Female, Medical Provider)

[The ASOs in the region] meet once a month. They will look at quality data. They will look at expenditures. They will look at service utilization data. They will talk about barriers. They will talk every single month about what’s going on in this community and what they can do and even down to expenditures where I can say “You know what, I have emergency financial assistance money in my organization and you know what, we’re going to run out. Does anybody have—?” “Oh, we have plenty. Why don’t you refer your clients over to us and we’ll take care of it for them.” (Participant 11, Male, Ryan White Administrator)

Coordination with the Department of Corrections was also crucial to HCC outcomes when PLWH transitioned back to the community, particularly for those with histories of substance use as is the case for many—if not the majority—of those incarcerated.

What gets you in jail, it’s—I mean there are some folks with violence charges and things like that, domestic and, but it’s mainly addiction that’s behind it, (Participant 31, Male, Medical Provider)

Participants noted that, prior to incarceration, many PLWH may have discontinued their HIV treatment. Incarceration represented an opportunity to resume HIV treatment that would be important to continue after release. Recognizing the high risk of relapse and potential need for post-incarceration substance abuse treatment, participants considered coordination of the responsibilities of parole/probation officers and case managers to be particularly critical to ensuring that the person remained in HIV treatment and did not return to prison due to probation/parole violation (which often involves illicit drug use).

We start working with them at least three months before they are released and then we assess what would be those challenges for them to stay in care once they are out of the jail. (Participant 36, Male, Case Worker)

When they come out of jail, I’ll go to probation and meet with them. I’ll talk to the probation. I have a drug counselor. I have a psychiatrist. I have primary care that we could help them. We maintain the medicines and once I tell them the plans that we got, the probation office is happy with us because we got a good relation with the probation officer because we’ll do their work [perform weekly urine screens, find the person]. (Participant 33, Male, Case worker)

Less frequently, participants reported that transfer of care was less than ideal in terms of notifying the ASO in a timely way of a prisoner’s release.

[The case worker] sometimes get notice that morning that so-and-so inmate is being released today, and they have to find a place for this person to go, and sometimes there’s no place for them to go. (Participant 1, Female, ASO administrator)

So we tested him at the jail. He got connected with some kind of service. I don’t believe he got connected with medical care there because he was going to leave and once he was out, he just disappeared until we found him months later. (Participant 40, Female, ASO administrator)

### Policy/society level themes

We identified two themes at the policy/society level: opposition and activities with unintended consequences.

#### Organizational and community opposition

The first addresses opposition in the form of policies or actions that could pose challenges to improving the HCC for at-risk populations such as those who use drugs or alcohol. The opposition could occur within an organization or within the larger community. The distinction is that organizations have unique internal procedures and contexts, and the larger community rarely has the opportunity to influence the organization’s decisions or policies. By contrast, community residents often can influence decisions about the budgets, programs, and policies of the local government.

Substance use-related visits to emergency departments (e.g., overdose/poisoning, trauma/falls while intoxicated, end-stage liver disease from alcohol use or hepatitis C infection) are not uncommon. Yet organizational opposition resulted in discontinuing routine, opt-out HIV testing in one hospital’s Emergency Department based on organizational doubt about the public health benefit of opt-out testing and desire to minimize potential bad publicity about legal problems during the pending sale of the hospital. This decision thereby decreased opportunities to identify cases among at-risk individuals who may have never been tested elsewhere.

The [new] director of the emergency room…just does not think this is the place for public health issues in [the] emergency room. That’s his attitude and you shouldn’t be doing [HIV testing] here…I literally just came from a meeting an hour and a half ago and they said “We’re not interested at this hospital in pursuing that right now.”…[the hospital is] in the middle of some legal stuff going on with that right now…the hospital is [also] being bought by another company and we’re getting to the closing and [the hospital is] just not interested in any bad publicity right now, so it’s not a good time for me to be talking about [routine HIV testing] in our emergency room, but it’s still something I’m very excited about. (Participant 3, Male, Medical Provider)

Despite the strong empirical evidence that SSPs can improve HCC outcomes [31, 32, 34–36, 38], programs faced challenges in operating in certain cities because of community opposition to the program, perceptions that SSPs are no longer needed, or inability to sustain regular hours when the SSP van required maintenance.

[The city council said,] “And so why would needle exchange help, because it’s not helping that the [HIV] numbers are down. So because the hepatitis C numbers were up is what really got [the city council] to do something different. But otherwise they’d say, “Why do we need it? What do we need testing for? The numbers are down.” (Participant 14, Female, City Health Department)

[The SSP clients] go, “You’re not here,” and we go, “Yeah, we’re really sorry, but we couldn’t get the van out,” or sometimes the van, the van’s a vehicle, like a car, so everyone’s like, “I need another van,” because we need one to [substitute just like] you need two cars in a house to take one to the mechanic, so this thing has to go in and get oil changes, mechanic, go get gasoline, it’s not like it’s a structure that stands. (Participant 33, Male, Case worker)

#### Activites with unintended consequences

Finally, anti-crime efforts and attempts to “clean up” high-risk neighborhoods resulted in the unintended consequence of increasing barriers to HIV testing and linkage efforts, particularly for people who inject drugs and men who have sex with men.

Well, a lot of abandoned buildings have been torn down. They redesigned the bus terminal and there’s policing out there, so there’s not a lot of people just hanging out anymore, same as the train station. So [activities of men who have sex with men have] gone more underground. Same as the drug use. It’s gone more underground. (Participant 30, Female, ASO administrator)

## Discussion and conclusions

The HCC strengths and successes noted by participants in the nine cities were many, although the 90-90-90 goal of the Joint United Nations Programme on HIV/AIDS [3] was not yet met by any of the ASOs participating in this study. ASO administrators were quick to note steady improvement in HCC outcomes over time, however. The most substantial HCC challenges noted were in linking and retaining HIV patients with multiple co-morbidities (substance use disorder, hepatitis C viral infection, mental health problems) and essential needs such as housing, food security, and transportation. These findings were consistent with previous studies and were thought to occur at all levels of the modified social ecological model [16, 19, 52–54].

At the individual level, per those interviewed, PLWH with ongoing or relapses in drug or alcohol use often had chaotic lives that frequently led to discontinuation of HIV treatment. These observations were consistent with the literature. Alcohol use was associated with poor HIV health outcomes [55–57]. Drug use often increased in response to tendencies to cope with receiving a diagnosis of HIV infection [58]. Expanding the availability of and easy access to effective drug and mental health treatments is recommended to reduce risk of delaying or discontinuing HIV treatment. The lives of PLWH with substance use problems could be further stabilized by ensuring secure living conditions (e.g., housing, food security, transportation) [59]. In addition, encouraging clients to actively engage in decisions about their care and to connect with other HIV consumers could increase opportunities for socioemotional support and promote a sense of community that may be particularly important for those whose social capital is largely confined to fellow substance users.

At the interpersonal level, it is well known that developing and maintaining positive client-staff relationships is essential to the successful provision of any healthcare or social service [60, 61] and was noted as essential to improving HCC outcomes in this and other studies [58]. Cultural competence and a non-judgmental, harm reduction approach were key to positive interactions, particularly for clients with internalized or anticipated stigma as a result of their substance use. It is, therefore, recommended that all staff receive periodic training on harm reduction, de-stigmatizing, and culturally competent approaches to interacting with PLWH with substance use problems in order to limit the potential for negative interactions that could ultimately compromise client-staff relationships and increase risk of discontinuing treatment.

We identified several themes at the health care system and policy/society levels—levels that are less frequently addressed in HCC research [14, 25]. At the health care system level, many of those incarcerated had histories of substance use, and although they qualified for housing benefits as PLWH, the availability of this entitlement was limited. In addition, incarceration provided an opportunity to return many to HIV treatment. This return to treatment is important to sustain post-release by not only linking these individuals to HIV treatment but also to substance abuse treatment services when indicated. Hence, permitting ASOs, other social and health service providers, and the Departments of Corrections to easily share individual patients’ data (e.g., current HCC status, medical care, social service needs and benefits) would permit quicker identification of and a more coordinated effort to satisfying individuals’ service needs as well as improving HIV treatment adherence and retention rates. By obtaining consent from everyone at the time of HIV diagnosis and linkage, such information could be shared with the relevant parties. It is suggested that consents be routinely updated when changes in care and services occur. The goal would be for all systems to remain as flexible and responsive as possible to the individual PLWH’s personal needs and preferences while preserving confidentiality. Bureaucratic barriers made such efforts difficult at times, particularly with respect to housing and in cases where PLWH were about to be released from detoxification treatment, prison, or jail. These barriers can create challenges for the HCC steps of treatment retention and viral suppression.

Perhaps the most compelling structural issue noted at the health care system level was the importance of case workers and peer navigators in retaining clients in care or returning them to care [52, 58, 62], particularly for cases of relapse in substance use. Although funding concerns were noted more generally and went beyond the scope of the current analysis, ASO administrators and providers were acutely aware that fluctuations in funding negatively impacted staffing considerations and desires to expand peer-to-peer programs. Coupled with the evidence from modelling studies that treatment retention is critical to improving HCC outcomes [63, 64] and that PLWH who either have ongoing substance use or who relapse are often among the most difficult clients to retain [7, 9, 65], case workers and peers are central to maintaining or returning them to treatment [66, 67], and salaries for these individuals should be ensured at all times.

Another key issue for those interviewed was making a comprehensive array of services available under one roof (i.e., “one-stop shopping”). Consistent with other studies, these services should be convenient [25], acceptable to consumers, and effective in addressing their treatment needs [16, 68]. The data indicated that agencies worked, with little evidence of inter-agency competition, to optimize coordination of both health and social services as much as possible when “one-stop shopping” was impossible. For those with opioid and alcohol dependence, medication-assisted treatment has been demonstrated to be cost-effective [69] and improves HCC outcomes [11, 14, 70]. Although co-location of HIV and medication-assisted treatment services did not exist at any of the organizations included in this study, it may be particularly important and worth exploring, given participants’ assertions of the tight-knit nature and close proximity of many local community collaborations and in the interest of better serving PLWH and improving HCC outcomes.

At the policy/society level, the community usually has little influence on organizational opposition to internal policies or decisions; this is in marked contrast to that of its potential influence at the community level. Hence, it is crucial to maximize communities’ potential influence at this level by ensuring that local governments are transparent about policies and activities and are open to discussions about harm reduction and HIV and substance abuse treatment efforts that include not only the members of the general community but also those with substance use problems. In addition, attempts to “clean up” neighborhoods in at least one of the nine cities made public health outreach efforts to test, link, or return PLWH to treatment particularly difficult for hard-to-reach populations such as people who inject drugs and men who have sex with men. To mitigate the potential for negative collateral consequences resulting from such policy decisions, public health officials and consumers should be involved in the decision-making process.

All three states permit syringe purchase at pharmacies without a prescription [71] as part of efforts to reduce HIV incidence. Yet SSPs were not legally authorized in five of the nine cities at the time of this study, despite the proven effectiveness of SSPs in HIV prevention and as a resource for HIV testing [32, 34, 36, 38], access to social and health services [72–75], HIV treatment [31, 35], and referral to drug treatment for injectors and non-injectors alike [33, 37]. In addition, many SSPs offer opioid overdose prevention and response training and distribution of naloxone which can save lives of PLWH who use opioids. It is therefore recommended that SSPs be implemented or expanded as a means of continuing the low HIV incidence among people who use drugs. SSPs can play an integral role in improving HCC outcomes by facilitating access to HIV testing, diagnosis, and linkage to treatment. Expansion of SSPs is occurring at a relatively fast pace in Massachusetts, following new amendments to syringe access legislation. In 2016, new SSPs in Worcester, Brockton, Lawrence, Greenfield, and North Adams were authorized and have the potential to greatly improve HCC outcomes in these small cities and non-urban areas.

The study had several limitations. First, the qualitative data were collected only from experienced HIV providers and public health staff. Hence, it is unclear whether the experiences and perceptions of PLWH about what shapes HCC outcomes are consistent with those of the KIs interviewed in this pilot study. In a follow-up study, we will soon interview PLWH both in and out of treatment to clarify this issue. Second, it appeared that participants were more willing to discuss the strengths and successes of their programs rather than HCC barriers or challenges attributable to organizational limitations. However, thematic saturation was achieved, and we therefore assume that additional interviews would not have identified additional themes. It is hoped that such limitations and gaps in knowledge could be addressed in future interviews with PLWH. Finally, the qualitative findings and associated recommendations are based upon a limited number of interviews within each city and state. Future studies are necessary to determine if additional themes exist, whether any of these findings apply to other locations, and whether the suggested recommendations will improve HCC outcomes for PLWH with drug and alcohol problems.

In conclusion, HCC outcomes have improved over time, but challenges persist. For PLWH with substance use problems and reside in smaller cities, there is a critical need for increased availability of and access to medication-assisted treatment and mental health services. Ideally, these and other ancillary services should be co-located with HIV treatment services. Expansion of case management and peer navigation services is also recommended in order to improve HIV treatment retention and re-engagement for this population. Finally, the data suggest that improved communication and coordination of services are necessary to improve HCC outcomes for PLWH who use substances. These objectives may be more easily achieved in small cities.
